# Magnetic Resonance Imaging Versus Computed Tomography for Three‐Dimensional Bone Imaging of Musculoskeletal Pathologies: A Review

**DOI:** 10.1002/jmri.28067

**Published:** 2022-01-19

**Authors:** Mateusz C. Florkow, Koen Willemsen, Vasco V. Mascarenhas, Edwin H.G. Oei, Marijn van Stralen, Peter R. Seevinck

**Affiliations:** ^1^ Image Sciences Institute University Medical Center Utrecht Utrecht The Netherlands; ^2^ Department of Orthopedics University Medical Center Utrecht Utrecht The Netherlands; ^3^ Musculoskeletal Imaging Unit Imaging Center, Hospital da Luz Lisbon Portugal; ^4^ Department of Radiology and Nuclear Medicine Erasmus MC, University Medical Center Rotterdam Rotterdam The Netherlands; ^5^ MRIguidance BV Utrecht The Netherlands

**Keywords:** MRI, CT, comparative review, diagnostic imaging, joints, bone and bones

## Abstract

Magnetic resonance imaging (MRI) is increasingly utilized as a radiation‐free alternative to computed tomography (CT) for the diagnosis and treatment planning of musculoskeletal pathologies. MR imaging of hard tissues such as cortical bone remains challenging due to their low proton density and short transverse relaxation times, rendering bone tissues as nonspecific low signal structures on MR images obtained from most sequences. Developments in MR image acquisition and post‐processing have opened the path for enhanced MR‐based bone visualization aiming to provide a CT‐like contrast and, as such, ease clinical interpretation. The purpose of this review is to provide an overview of studies comparing MR and CT imaging for diagnostic and treatment planning purposes in orthopedic care, with a special focus on selective bone visualization, bone segmentation, and three‐dimensional (3D) modeling. This review discusses conventional gradient‐echo derived techniques as well as dedicated short echo time acquisition techniques and post‐processing techniques, including the generation of synthetic CT, in the context of 3D and specific bone visualization. Based on the reviewed literature, it may be concluded that the recent developments in MRI‐based bone visualization are promising. MRI alone provides valuable information on both bone and soft tissues for a broad range of applications including diagnostics, 3D modeling, and treatment planning in multiple anatomical regions, including the skull, spine, shoulder, pelvis, and long bones.

**Level of Evidence:**

3

**Technical Efficacy:**

Stage 3

Magnetic resonance imaging (MRI) is a radiation‐free, noninvasive imaging modality that provides three‐dimensional (3D) visualization of tissues. Its superior soft tissue contrast has made it a preferential diagnostic tool for the imaging of various organ systems, including the musculoskeletal system. Osseous structures are, however, usually visualized using radiography or computed tomography (CT). For imaging complex structures, CT is preferred as it offers high‐resolution 3D images with a radiodensity contrast that highlights bony tissues. Building upon the characteristic high X‐ray attenuation of cortical bone, dedicated (semi‐)automatic bone segmentation tools have been developed for CT images. Resulting 3D bone renderings have proven valuable in the diagnosis and treatment of bone pathologies. Consequently, pathologies affecting both soft and hard tissues, including skull,^1^, ^2^ spine,^3^, ^4^, ^5^ and joint disorders,^6^, ^7^ often warrant the acquisition of both MR and CT images. Such a multimodal workflow is logistically complex and induces an adverse radiation burden inherent to CT imaging, especially harmful in young population.^8^

Recent advances in MR image acquisition and processing, facilitated by the development of new hardware and the increase in computing power, have enabled the improvement of bone contrast on MR images. If reliable, MRI could be a radiation‐free alternative to CT for the diagnosis and treatment planning of certain musculoskeletal pathologies. Transforming a CT‐MR multimodal workflow into a simplified radiation‐free MR‐only workflow, as previously proposed in radiotherapy treatment planning,^9^ could lead to less hospital visits, lower costs, allow for the fusion of soft tissue and bone information, and reduce the time under sedation for younger patients.^10^, ^11^ This review will discuss comparative studies of MRI and CT for the diagnosis and treatment planning of bone pathologies in musculoskeletal diseases in multiple anatomical regions, including the skull, the spine, the shoulder, and the pelvis. Four main subjects will be described: MRI‐based techniques for bone imaging, MRI for bone segmentation and 3D reconstruction, MRI for the diagnosis of bone pathologies, and the remaining challenges faced by MRI in the context of bone visualization. Applications in the fields of radiotherapy and positron emission tomography–magnetic resonance (PET‐MR) will not be covered as they have been thoroughly reviewed in the past few years.^12^, ^13^, ^14^, ^15^

## MRI‐Based Visualization of the Bone Morphology

**FIGURE 1:**
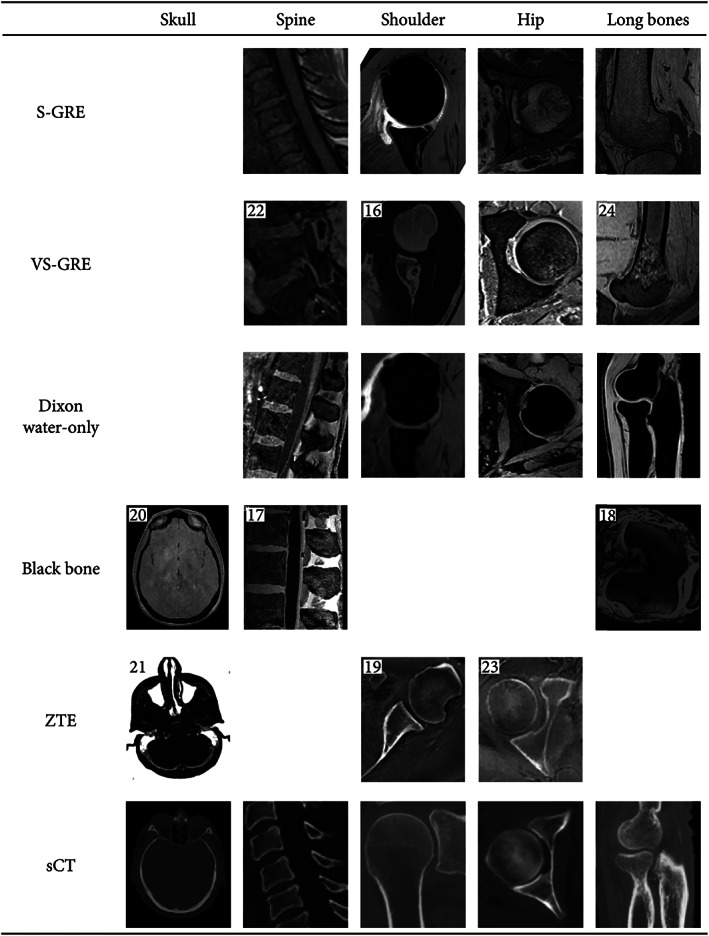
Illustrations of the various MR contrasts used for bone visualization in multiple anatomies. Some of the images were fat‐suppressed (eg, S‐GRE of the shoulder) or post‐processed (eg, VS‐GRE of the shoulder). Some images are reprinted with permissions from the reference given in the top left‐hand corner of the images.^16^, ^17^, ^18^, ^19^, ^20^, ^21^, ^22^, ^23^, ^24^ Black bone/skull was reprinted by permission from Springer Nature.^20^ Original images were all cropped to only show the region of interest. MR = magnetic resonance; sCT = synthetic computed tomography; S‐GRE = radiofrequency spoiled gradient‐echo; VS‐GRE = volumetric radiofrequency spoiled gradient‐echo; ZTE = zero echo time.

**FIGURE 2:**
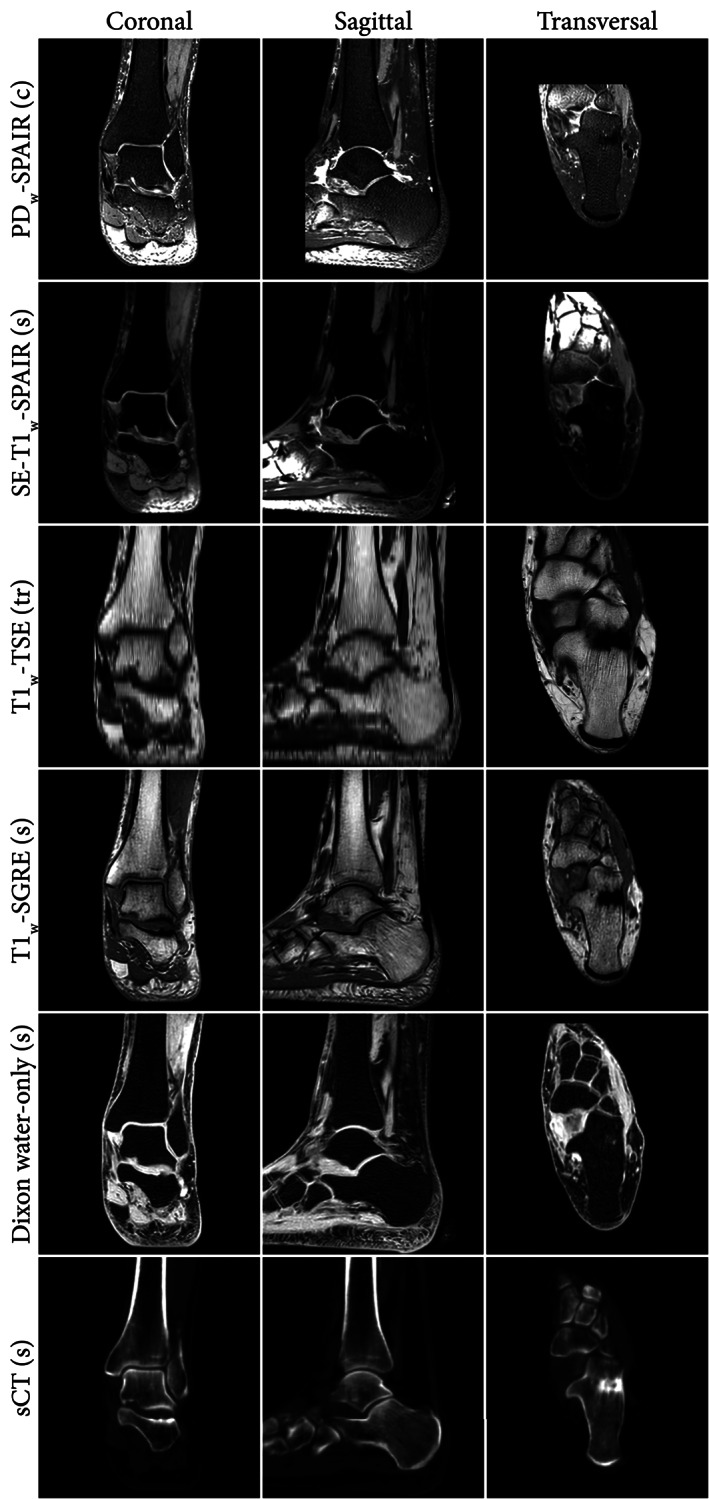
Example coronal, sagittal, and transversal slices of the same anatomical region obtain from MRI. The letters between brackets indicate the acquisition plane (c: coronal, s: sagittal, tr: transversal). Dixon water‐only and synthetic CT (sCT) images were based on the T1w‐S‐GRE image. PDw = proton density‐weighted; T1w = T1‐weighted; SPAIR = spectral attenuated inversion recovery (fat suppression); SE = spin‐echo; TSE = turbo spin‐echo; S‐GRE = radiofrequency spoiled gradient‐echo; MRI = magnetic resonance imaging.

Cortical bone imaging is challenging with MRI due to its low free‐water content. The MR signal that originates from cortical bone is mostly emitted by bound water, causing the signal decay to be rapid. Consequently, in conventional MRI sequences, cortical bone appears as a structure with low signal intensity that is not specific to bone. Although valuable for structural imaging, the poor visualization of cortical bone on conventional sequences has motivated the development of dedicated imaging techniques that facilitate bone visualization and segmentation. The remainder of this section briefly discusses several MR sequences and processing techniques used for bone imaging. The resulting MR images are compiled in Fig. 1 which provides an overview of multiple anatomical regions and in Fig. 2 which displays ankle images of a single patient. For each sequence, Table 1 provides the reported acquisition parameters, and Table 2 summarizes their characteristics and fields of study.

**TABLE 1:** Acquisition Parameters of the MR Sequences Reported in This Review

Sequence Type	Anatomies	Field Strength (T)	Resolution (mm)	FOV (mm)	Acquisition Time	TE	TR	Flip Angle (°)	Bandwidth
Dixon‐T1w‐GRE	Hip,^25^ shoulder^26^	1.5	**0.9 × 0.9 × 0.9**	288 × 288	7 minutes 33 seconds	2.4/4.8	7.4	9	490 Hz/px
Dixon—S‐GRE	Hip^27^	1.5	**0.6 × 0.6 × 0.67** ^a^	270 × 270 × 120	4 minutes 38 seconds	2.0/4.3	6.7	20	541 Hz/px
	Hip^27^	3	**0.97 × 0.97 × 1** ^a^	435 × 435 × 160	2 minutes 38 seconds	2.1/3.5	6.5	10	1122 Hz/px
	Hip^28^	3	**1 × 1 × 1** ^a^	200	3 minutes 28 seconds	2.4/?	10	145	350 Hz/px
	Shoulder^29^	3	**1 × 1 × 1** ^a^	200	3 minutes 28 seconds	2.4/3.7	10	10	400 Hz/px
Dixon—VS‐GRE	Hip^30^	3	1.2 × 1.2 × 1	175 × 320 × 192	32 seconds	1.27/2.5	3.9	9	NA
S‐GRE	Long bone^31^	1.5	0.45 × 0.45 × 1	NA	20 minutes	4.9	11	15	NA
	Long bone^32^	1.5	0.39 × 0.39 × 1^a^	200 × 200^b^	NA	4	17	25	NA
	Long bone^33^	1.5	0.39 × 0.39 × 1^a^	NA	NA	5	20	12	NA
	Shoulder^34^	1.5	**0.7 × 0.7 × 0.7**	NA	NA	5.1	23	50	NA
	Shoulder^35^	1.5	0.31 × 0.625 × 1^b^	160 × 160	3.5 minutes	4.38	1290	15	NA
	Shoulder^35^	3	0.54 × 0.68 × 1.5^b^	100 × 100	3.5 minutes	2.3	5.1	30	NA
	Hip^30^	3	**1 × 1 × 1**	170 × 170	16 minutes (3 scans)	3.3	15	4/24	NA
	Spine^36^	3	0.28 × 0.28 × 0.75^a^	70 × 160 × 250	5 minutes 7 seconds	2.3	7.8	8	NA
	Spine^37^	3	0.94 × 0.94 × 2^a^ ^,^ ^b^	240	6 minutes 43 seconds	7	27	30	20.8 kHz
VS‐GRE	Long bone^38^	3	0.31 × 0.31 × 0.7	160 × 160	3 minutes 34 seconds	5.4	11.6	NA	NA
	Long bone^24^	3	0.47 × 0.47 × 1	240 × 120	9 minutes 37 seconds	1.83	11	NA	NA
	Spine^39^	3	? × ? × 2	200	4 minutes	2.45	7	NA	NA
	Spine^22^	1.5	0.94 × 2.4 × 1.7	240	NA	2.4	6.52	NA	NA
	Shoulder^16^	3	**0.7 × 0.7 × 0.7**	180 × 180	4 minutes 16 seconds	3.5	10.5	12	300 Hz/px
	Shoulder^7^	3	**0.9 × 0.9 × 0.9**	180	<2 minutes	4.9	12.3	10	NA
	Shoulder^40^	3	**0.6 × 0.6 × 0.6**	160 × 160	3 minutes 14 seconds	4.9	12.2	10	NA
	Shoulder^41^	3	**0.82 × 0.82 × 0.89**	210 × 210 × 100	5 minutes	2.66	7.16	10	300 Hz/px
	SI joint^6^	1.5	? × ? × [0.89–1]	NA	3–5 minutes	7	25	NA	NA
	SI joint^42^	3	**0.6 × 0.6 × 0.6**	154 × 154^b^	3 minutes	5.2	11.7	10	NA
	Hip^43^	1.5	**1 × 1 × 1**	NA	4 minutes	2.88	7.67	NA	NA
	Hip^44^	3	**0.8 × 0.8 × 0.8**	160 × 160 × 102^b^	9 minutes	3.3	15	4 and 24	NA
	Hip^45^	3	**0.8 × 0.8 × 0.8**	180 × 340	6 minutes 33 seconds	4.9	10.8	NA	NA
GRE	Long bone^46^	0.3	0.39 × 0.39 × 1	160	11–15 minutes	NA	NA	NA	NA
	Long bone^47^	1.5	0.39 × 0.39 × 0.7^a^	200 × 200	NA	NA	NA	NA	NA
	Long bone^48^	3	0.3 × 0.3 × 0.5	[120–150]	5 minutes 30 seconds to 6 minutes 45 seconds	2.7	10	8	NA
	Skull^49^, ^50^	1.0	**0.9 × 0.9 × 1** ^a^	230 × 230^b^	NA	6.9	25	NA	NA
UTE	Long bone^51^	3	0.25 × 0.25 × 2	400	7 minutes	0.032	100	10	NA
	Skull^52^	1.5	0.5 × 0.5 × [1, 2]^a^ ^,^ ^b^	256 × 256 × 140	[6–8] minutes	[0.08–0.35]/[2.3–4.6]	[8–12]	25	NA
	Skull^53^	3	**0.7 × 0.7 × 0.7**	246	5 minutes 15 seconds	0.07	5	NA	355 Hz/px
	Skull^54^	3	**1.1 × 1.1 × 1.1**	280 × 280 × 280	6 minutes	0.06/2.46	7	12	NA
	Spine^36^	3	0.28 × 0.28 × 0.75^a^	279 × 259 × 250	6 minutes 18 seconds	0.14	6.3	5	NA
	Spine^37^	3	0.94 × 0.94 × 2^b^	240	3 minutes 12 seconds	0.05	44.3	2	125 kHz
	Spine^55^	3	0.8 × 0.8 × 1.2	230 × 230 × 119	8 minutes 56 seconds	0.2/4.6	10.2	10	NA
	Shoulder^56^	3	0.83 × 0.83 × [3, 4]	160	3–4.5 minutes	0.03	134	18	NA
ZTE	Skull^57^	3	0.69 × 0.69 × 1^b^	180 × 180	~5 minutes	0	785	4	31.25 kHz
	Skull^21^	3	**0.85 × 0.85 × 0.85**	220	6 minutes 12 seconds	0	NA	1.2	62.5 kHz
	Skull^21^	3	**1.35 × 1.35 × 1.35**	260	2 minutes 53 seconds	0	NA	1.2	62.5 kHz
	Skull^58^	3	**1.4 × 1.4 × 1.4**	260 × 260 × 260	2 minutes 52 seconds	0	NA	1	62.5 kHz
	Spine^5^	3	**1 × 1 × 1**	320	~5 minutes	0	417	1	62.5 kHz
	Shoulder^59^	1.5/3	[0.625–1.2] × [0.625–1.2] × 1.4	[200–300]	4–6 minutes	[25–40]	[1.375–1.629]	1	62.5 kHz
	Shoulder^19^	3	**1 × 1 × 1**	200	3–4 minutes	0	[0.8–1.1]	1	62.5 kHz
	Shoulder^19^	3	**0.7 × 0.7 × 0.7**	200	10–13 minutes	0	[0.8–1.1]	1	62.5 kHz
	Hip^23^	3	**~[1.1–1.4] × [1.1–1.4] × [1.1–1.4]** ^a^	[360–440]	5 minutes	0	[425–528]	1	62.5 kHz
BB‐MRI	Long bone^18^	3	**1 × 1 × 1**	401 × 401	10 minutes	3.69	10	5	NA
	Skull^20^, ^60^, ^61^, ^62^	1.5	0.94 × 0.94 × 1.2^b^	240	4 minutes	4.2	8.6	5	31.25 kHz
	Skull^63^	3	0.47 × 0.47 × ?^a^ ^,^ ^b^	240	7 minutes 50 seconds	2.2	6.7	5	610 Hz/px^b^
	Skull^64^	3	1.6 × 1.6 × 3	? × ? × 240	~3 minutes	[1.51–1.55]	[3.56–3.62]	12	[590–610] Hz/px
	Spine^17^	3	0.625 × 0.325 × 0.6^b^	240 × 144	6 minutes	3.1	7.4	NA	62.5 kHz
BB‐MRI‐VS‐GRE	Skull^64^	3	**1 × 1 × 0.9**	192 × 192	~3 minutes	In‐phase	25	3 and 5	[590–610] Hz/px
BB‐MRI‐UTE	Skull	1.5/3	**1.1 × 1.1 × 1** ^b^	250 × 250	4 minutes	0.07	3.32	NA	NA
Spin‐echo derived	Skull^65^	3	0.53 × 0.53 × 0.53	171 × 171	7 minutes 1 second	5.8	800	NA	625
	Spine^22^	1.5	0.87 × 3.5 × 3	280	NA	19	624	NA	NA

In red, acquisition time longer than 5 minutes. In bold, nearly isotropic acquisition. Question mark and NA indicate the information was not available.

GRE = gradient‐echo; S‐GRE = radiofrequency spoiled gradient‐echo; UTE = ultrashort echo time; ZTE = zero echo time; BB = black bone; FOV = field of view; TE = echo time; TR = repetition time; MRI = magnetic resonance imaging.

^a^ Indicates this is the reconstructed resolution.

^b^ Indicates that the parameter was not directly given and computed using other parameters reported in the manuscript.

**TABLE 2:** Characteristics and Fields of Applications of Multiple MR Acquisition and Processing Techniques

	GRE	BB	UTE	ZTE	sCT
Generation	Acq.	Acq.	Acq.	Acq.	Processing
Hardware requirement	Low	Low	High	High	Low
Software requirement	Low	Low	High	High	High
Availability	High	High	Low	Low	Medium
Pros	‐ Fast 3D (isotropic) acquisition	‐ Uniform soft tissue contrast	‐ Acquisition of cortical bone signal	‐ Fast 3D isotropic silent acquisition ‐ Not sensitive to motion ‐ Acquisition of cortical bone signal	‐ Quantitative (Hounsfield units)
Cons	‐ Low signal acquired in cortical bone	‐ Low signal acquired in bone	‐ Susceptible to gradient inhomogeneity ‐ Spatially nonselective 3D excitation	‐ Spatially nonselective excitation	‐ Questionable reliability
Applications	‐ Fracture detection ‐ Structural changes ‐ Morphometric parameters assessment	‐ Fracture detection ‐ Skull suture assessment ‐ Surgical planning	‐ Structural changes ‐ Fracture detection	‐ Structural changes ‐ Morphometric parameters assessment	‐ Structural changes ‐ Morphometric parameters assessment ‐ Surgical planning
Anatomical regions	‐ Skull ‐ Spine ‐ Shoulder ‐ Hip ‐ Long bones	‐ Skull ‐ Spine ‐ Long bones	‐ Skull ‐ Spine ‐ Shoulder ‐ Long bones	‐ Skull ‐ Spine ‐ Shoulder ‐ Hip	‐ Spine ‐ Pelvis ‐ Long bones

GRE = gradient‐echo; BB = black bone; UTE = ultrashort echo time; ZTE = zero echo time; sCT = synthetic computed tomography; Acq. = acquisition; MR = magnetic resonance.

### Conventional Clinical MR Sequences

**FIGURE 3:**
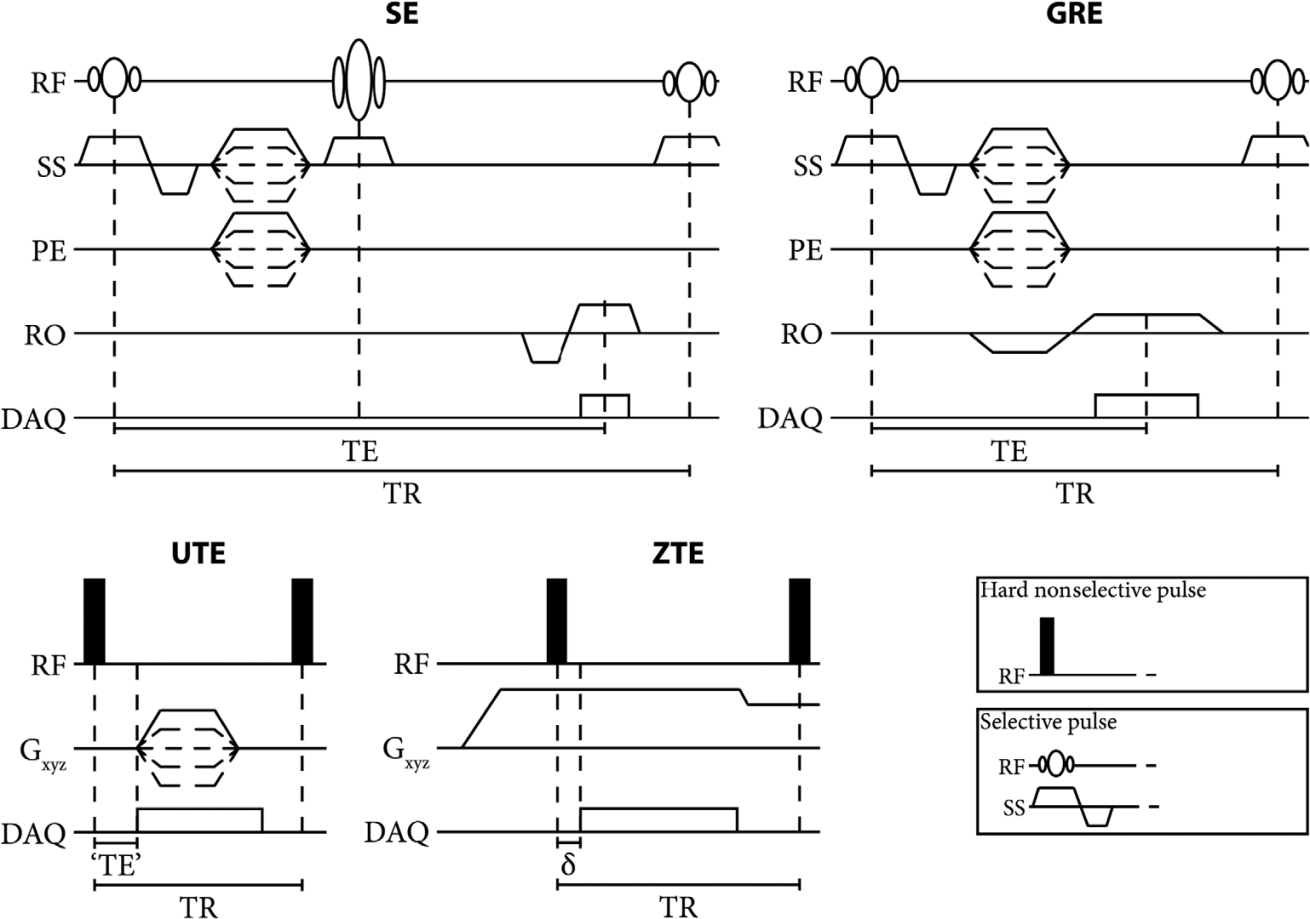
Chronograms of basic spin‐echo (SE) gradient‐echo (GRE), ultrashort echo time (UTE), and zero echo time (ZTE) pulse sequences. Note the difference in echo time (TE) and repetition time (TR) between the sequences. Typical values of TE in the UTE sequence are in the range of 100 μs and of δ in the ZTE sequence in the range of 10 μs. In particular, in the UTE sequence, there is a fast gradient switching between TR's and the acquisition starts during the gradient ramp up. In the ZTE sequence, gradient switching is smooth and the gradient is on before the excitation but there is a delay between the excitation and the acquisition. In this basic UTE sequence, a free induction echo is acquired but more complex sequences can acquire gradient‐recalled echoes. RF = radiofrequency; SS = slice selection; PE = phase encoding; RO = readout; DAQ = data acquisition.

Within the field of musculoskeletal imaging, T1‐ and proton density (PD)‐weighting is often acquired for structural bone imaging whereas T2‐weighting is acquired for imaging functional and pathophysiological processes. T1‐weighted (T1w) images have been acquired to detect structural lesions using spin‐echo (SE) or gradient‐echo (GRE) sequences. SE images and their derivatives are routinely acquired in musculoskeletal radiology owing to their excellent soft tissue contrast. Compared to GRE, SE sequences are also less prone to susceptibility, chemical shift, and field inhomogeneity artifacts but are nonetheless affected by geometrical distortions, especially at low receiver bandwidth and in regions far from the bore isocenter.^66^ On the other hand, GRE sequences are usually faster owing to a shorter minimal repetition time (TR) as shown in the pulse sequence chronograms in Fig. 3. In addition, GRE sequences are more versatile and are increasingly investigated for musculoskeletal radiology using radiofrequency spoiled gradient‐echo (S‐GRE also known as vendor‐specific acronyms FLASH [fast low angle shot], SPGR [spoiled gradient‐recalled], or T1‐FFE [T1 fast field echo]), or volumetric radiofrequency spoiled gradient‐echo (VS‐GRE also known as vendor‐specific acronyms VIBE [volumetric interpolated breath‐hold examination], LAVA [liver acquisition with volume acquisition], or THRIVE [T1‐weighted high‐resolution isotropic volume examination])^67^ that enable post‐acquisition multiplanar reformatting.

For bone morphology visualization, these sequences have been proposed in combination with fat‐saturation^37^, ^39^, ^47^ or water excitation^16^ to suppress the signal from adipose tissues and render bone as structures with a uniform low intensity. Alternatively, water‐only images can be generated by acquiring a GRE sequence with specific echo times to perform a Dixon water–fat separation.^25^, ^28^, ^29^, ^68^, ^69^

### Dedicated MR Sequences

To further improve bone visualization, sequences have been developed to enhance bone specificity by providing a uniform soft tissue contrast, or by aiming at a CT‐like contrast.

#### BLACK BONE IMAGING

The gain in bone specificity was achieved in “black bone” (BB) sequences by applying a low flip angle and short echo time (TE) and TR to GRE‐like sequences,^60^ including VS‐GRE^64^ and ultrashort echo time (UTE)^70^ sequences. With such parameters, cortical bone appears as a low‐intensity structure whereas soft tissues have a uniform intermediate intensity. Originally developed for craniofacial imaging,^60^ BB‐MRI has been further applied to image the spine,^17^, ^71^ and long bones.^18^

#### ULTRASHORT AND ZERO ECHO TIME IMAGING

Subsequently, the development of new hardware, which enabled faster transmit/receiving switching coils and more demanding gradients, permitted a drastic lowering of the echo time resulting in UTE sequences. In such sequences, the signal is usually acquired radially, soon after the end of the excitation, before a T2‐induced signal decay and minimal T2* signal decay. An image with a CT‐like contrast containing signal mainly in short T2 components can then be obtained by suppressing the long T2 signal.^72^ However, the fast gradient switching between TR's and the acquisition during the gradient ramp up (Fig. 3) renders UTE sequences prone to Eddy currents and susceptible to gradient delays, potentially resulting in imaging artifacts.

With further developments, zero echo time (ZTE) images have been acquired for which the signal is sampled (usually radially) directly after the application of the radiofrequency (RF) pulse. To that end, readout gradients are on during the RF excitation. However, because of the delay in switching from transmit to receive modes, there is a dead time during which the center of the k‐space is not sampled (Fig. 3). Consequently, to reduce the dead time, hard short RF pulses need to be used, which put constraints on the achievable flip angles and bandwidths. ZTE images are acquired only with free induction decay readout, and a CT‐like contrast can be obtained by applying an inverse‐logarithmic rescaling.^72^ Since gradient switching is smooth (Fig. 3), ZTE acquisitions are rather silent, and the short achievable TR makes them fast.

### Image Processing Techniques

In addition to the advances in image acquisitions, image analysis and processing techniques have been applied to enhance bone visualization, usually aiming to create images with a CT‐like contrast. The simplest processing steps consisted in inverting the intensities or subtracting water intensities from the entire image, thus highlighting low signal in the MR images which is hypothesized to reflect the presence of cortical bone. Such a technique has been applied on standard GRE,^48^ VS‐GRE,^16^ or Dixon water‐only images.^26^, ^29^ More advanced processing has been searched to convert MR image intensities to CT Hounsfield units (HU), creating so‐called synthetic CT (sCT). The most promising sCT generation models are deep learning‐based and rely on various network architectures, including UNet,^73^ generative adversarial network,^74^ and their derivatives.^75^ The use of sCT images has already been reviewed multiple times for radiotherapy purposes and PET‐MR^9^, ^12^, ^13^, ^14^ but their use for orthopedic purposes is rare. sCT generation models for orthopedic care have mainly been developed for the pelvis,^27^, ^76^, ^77^ sacroiliac joint,^78^ spine,^79^, ^80^ and long bones.^81^, ^82^

## MRI for Three‐Dimensional Bone Modeling

Three‐dimensional bone renderings are gaining popularity in orthopedic care as they provide an overview of the bone morphology, enable kinematic analyses,^30^, ^41^ and allow for the patient‐specific design of surgical guides and implants.^63^, ^82^ Hence, 3D bone models facilitate the clinical diagnosis and improve surgical outcomes,^26^, ^43^, ^70^, ^83^, ^84^, ^85^ motivating their use in the treatment management of pathologies in the skull, shoulder, and hip.^43^, ^83^, ^84^, ^85^ Therefore, to be a CT surrogate for bone visualization, MRI should provide images on which bone can be segmented within a time and with a level of accuracy similar to or better than what can be achieved on CT. This section describes approaches for bone segmentation on MRI and provides results on segmentation geometrical accuracy and segmentation time with applications related to surgical planning.

### Bone Segmentation

**FIGURE 4:**
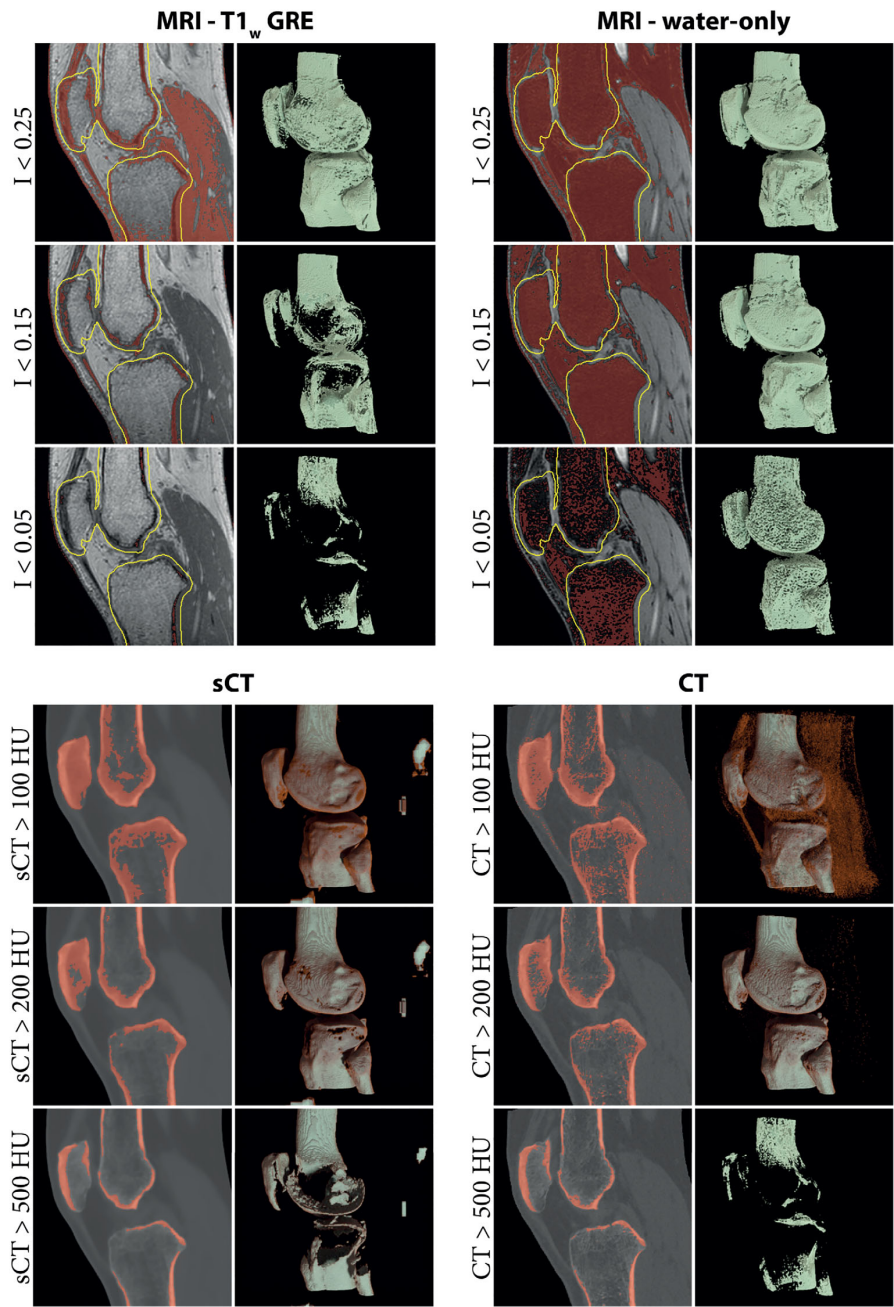
Bone segmentation and corresponding renderings obtained through the application of a simple threshold on magnetic resonance imaging (MRI) and computed tomography (CT) images. The low signal on acquired MR images is not specific to bone and other structures are included in the segmentation when thresholding is used alone. Therefore, for the T1‐weighted gradient‐echo (T1w‐GRE) and water‐only images, renderings were computed only in a region of 5 mm around the ground truth bones (yellow line) to focus on the bony region and hide most segmented soft tissues. Synthetic CT (sCT) images, by representing Hounsfield units (HU) enable a quick segmentation of bone, similar to what can be obtained on CT.

Regardless of the acquired MR contrast, there is a lack of MR‐dedicated, automated software for bone segmentation as exists for CT images. Bone segmentation on MR is mainly manual, or with extensive manual editing,^16^, ^17^, ^25^, ^28^, ^29^, ^30^, ^33^, ^41^, ^47^, ^54^, ^70^, ^86^ although some (semi‐)automated methods based on thresholding,^7^, ^26^ region growing,^32^ or ray casting^19^ can be applied. The development of fully automated segmentation approaches is complicated by structures in the vicinity of bones that share the same intensity as (cortical) bone and that can consequently be wrongly included in the bone segmentation. The problematic anatomical areas depend on the acquisition sequence but usually include air,^54^, ^58^, ^70^ and soft tissues like tendons, ligaments, or labrum.^7^, ^31^, ^81^ sCT images are a special case for segmentation as they are quantitative and reproduce HU from CT images. Hence, sCT can benefit from HU‐based segmentation and CT‐dedicated software^27^, ^81^, ^82^ as demonstrated by the segmentations of the knee bones obtained from S‐GRE, Dixon water‐only, sCT, and CT images in Fig. 4.

**FIGURE 5:**
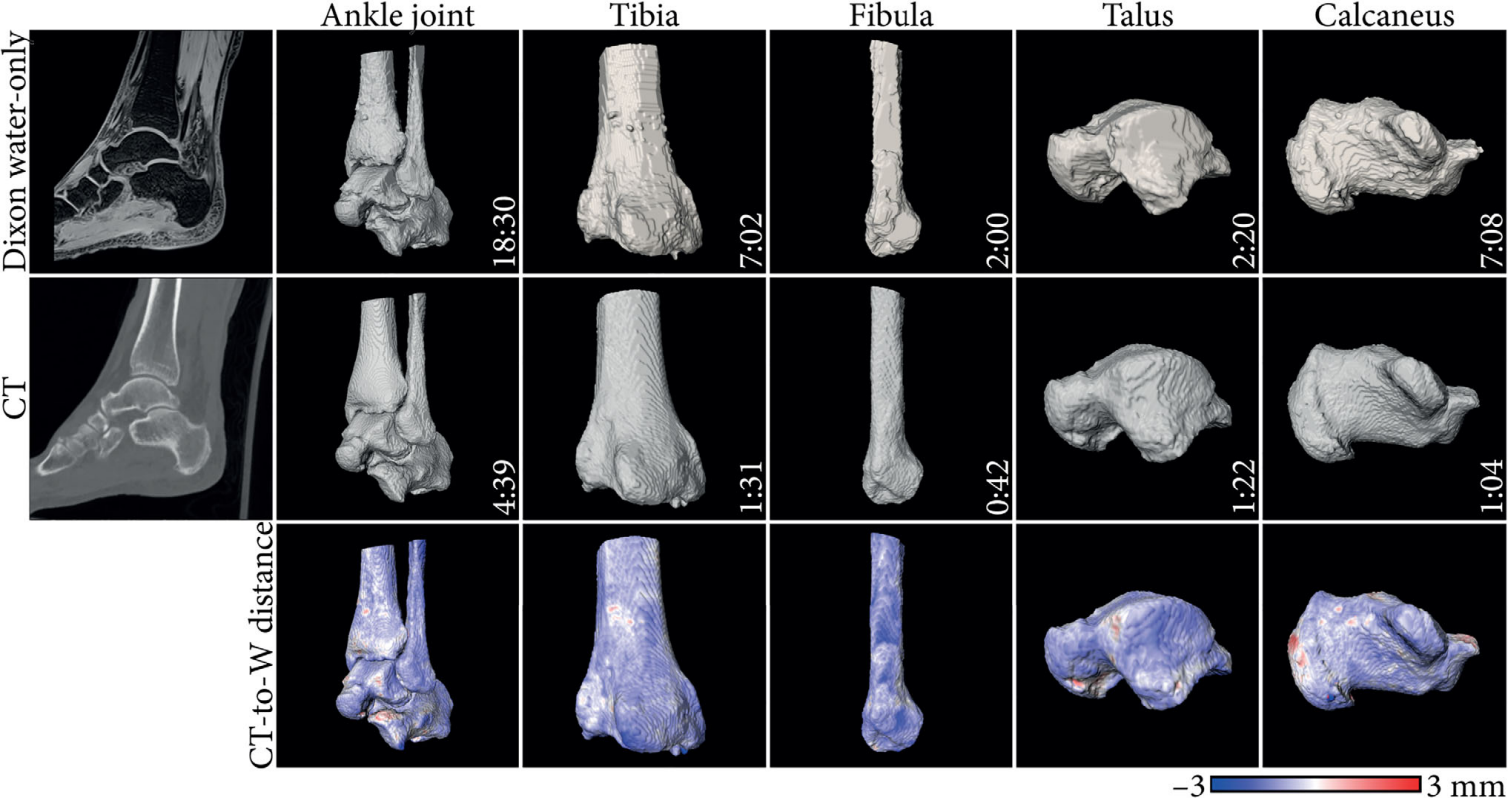
Example segmentations obtained from bone Dixon reconstructed water‐only (W) and computed tomography (CT) images of the ankle joint. Segmentations were performed by a junior engineer using Slicer 4.11 (https://www.slicer.org/). The time required to perform the segmentation is reported in the format mm:ss. Surface distance maps from CT to magnetic resonance imaging (MRI)‐based bone renderings are displayed. Negative values indicate the CT‐based segmentation is larger.

In total, MR segmentation of bone lasted from 33 seconds to 5 hours^16^, ^19^, ^29^, ^32^, ^47^ in the reported literature and were made on standard of care fat‐suppressed MR,^47^ GRE‐MR,^86^ S‐GRE,^31^, ^32^, ^33^, ^46^ VS‐GRE,^7^, ^30^, ^41^ processed GRE‐derived,^16^, ^25^, ^26^, ^28^, ^29^, ^43^ BB,^17^, ^18^, ^54^, ^63^, ^70^ ZTE,^19^, ^59^ and sCT^27^, ^77^, ^81^, ^82^ images. This duration depends on the anatomy, the user's experience,^16^, ^19^, ^30^, ^86^ the segmentation method, and the desired quality of the segmentation, which hinders comparisons between studies. However, compared to CT‐based segmentation within the same study, segmentation on MR images was usually more time‐intensive,^16^, ^19^, ^29^, ^54^, ^86^ sometimes requiring more than twice the time.^30^, ^43^, ^47^ As an example, Fig. 5 presents timed segmentations of ankle bones obtained from CT and Dixon water‐only images. Nevertheless, when the segmentation was done by experts or companies, no difference was noted in segmenting bone from MR or CT images in terms of processing time.^19^, ^63^ To alleviate the impact of user's experience on bone segmentation, automated methods based on deep learning are being developed^44^, ^87^ and are becoming commercially available for limited applications (eg, Mimics Innovation Suite 24, Materialize, Leuven, Belgium or CoLumbo, SmartSoft, Varna, Bulgaria).

### Bone Geometrical Accuracy

Regions prone to motion or magnetic field inhomogeneity could compromise the geometrical accuracy of the bone as seen on MR images. The geometrical integrity of the image can also be altered by nonlinear encoding gradients that may introduce compression or stretching of parts of the image. Because geometrical distortions could alter bone morphology, and consequently MR diagnostic capabilities, the overall geometry of the bone as visualized using MRI has been compared to physical ex vivo specimens and in vivo to CT, which is reviewed below.

#### COMPARISON TO BONE CADAVERIC SPECIMENS

The geometrical accuracy of bone segmentation has been evaluated on long bones and vertebrae in ex vivo studies so that MR segmentation could be compared to the physical bone shape using 3D printing,^47^ mechanical contact/optical scanners,^18^, ^31^, ^32^, ^33^ or micro‐CT.^31^, ^82^ Bone specimens were processed to remove soft tissues, resulting in a potential shrinkage of the gold standard compared to the bone as scanned using MRI and CT.^18^, ^25^, ^33^, ^82^

On average, CT segmentation overestimated the actual bone shape, whereas MR segmentation mostly underestimated it,^31^, ^32^, ^33^, ^47^ although not consistently.^25^, ^82^ Nevertheless, surface distances between the MR‐based segmentation and the cadaveric specimen were on average submillimeter,^17^, ^25^, ^31^, ^33^, ^82^ with mean absolute surface distances ranging from 0.23 mm to 0.41 mm for MR‐segmentations and from 0.15 mm to 0.51 mm for CT‐based segmentations.^17^, ^25^, ^31^, ^33^ Similarly, root mean square error (RMSE) was mainly submillimeter,^18^, ^31^, ^33^ although it could reach 1.2 mm^32^ in the knee for MRI models (vs. 0.5 mm for CT models).

When the CT‐based segmentation was used as a reference, the MR‐based bone segmentation also showed a submillimeter accuracy. In ex vivo long bones, absolute surface distances ranging from 0.23 mm to 0.61 mm were reported,^31^, ^38^, ^81^ with limits of agreement of the signed surface distance within ±0.72 mm,^18^ and RMSE of 1.1 mm.^88^ In ex vivo skulls, which can be harder to register and segment, BB‐MRI segmentations deviated on average by ±1.4 mm from CT segmentations.^63^

Larger differences were generally observed between the reference and MRI models near the joints in the proximal and distal bone ends,^31^, ^82^ although not always with statistical significance.^18^ Such differences resulted from the multiple soft tissues present at these locations (muscle, tendons, cartilage, and ligaments) which induce partial volume effects that hinder bone segmentation and warrant manual editing.^31^, ^81^ Alternatively, errors were observed at the edge of the field of view (FOV), where there is less signal.^33^

#### COMPARISON TO CT‐BASED BONE SEGMENTATION IN VIVO

The average submillimeter accuracy of bone segmentations compared to bone specimens shows that MR images have the ability to provide geometrically accurate bone models. To take into account soft tissue, evaluate more complex anatomies, and to make comparisons in an in vivo setting, MR segmentations were compared to CT segmentations. In in vivo hip joints, MR bone models differed on average by 0.4–0.9 mm from CT models,^27^, ^30^ with average RMSE under 1.8 mm^30^ for VS‐GRE Dixon images and under 0.81 mm for sCT images.^77^ When considering in vivo knees, there was no difference in the width and volume of the medial tibial plateau, with highly consistent measurements between standard of care PDw MR and CT images.^89^

This geometrical accuracy was influenced by the MR sequence acquired to perform the segmentation. Compared to other MR sequences, VS‐GRE offered the best correspondence to CT in the knee, with up to 45% differences in surface distance between VS‐GRE and balanced steady‐state GRE or spin‐echo derived images.^38^ VS‐GRE sequences had a better soft‐tissue‐to‐bone contrast, offering easier and more reproducible segmentations. Unfortunately, studies did not often report on registration parameters and acquisition parameters, such as the receiver bandwidth (see Table 1) or the built‐in distortion correction that can affect surface distance measurements and geometrical accuracy,^66^ preventing further comparisons between sequences and studies.

### Surgical Planning

Bone models obtained from segmentations can serve as a diagnostic tool in the therapeutic decision‐making, but also for surgical planning by allowing the design of customized surgical guides and implants.

**FIGURE 6:**
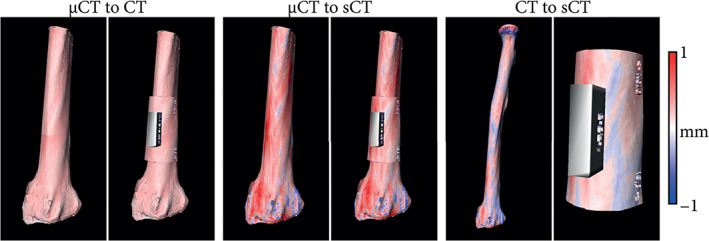
Example of an ex vivo radius used for evaluating different modalities for osteotomy planning. Bone renderings were generated from micro‐CT (μCT), CT, and synthetic CT (sCT) images and were used to design saw guides in identical locations. The color map indicates the surface distance between the bone renderings/saw guides obtained from the different modalities. Negative values indicate the μCT/CT is larger.

In the lower arm, saw guides for osteotomy were designed from CT and MR‐based sCT images and placed on cadaveric bones.^82^ The average saw guides positioning errors compared to the virtual planning were 2.4 mm and 3.8° for CT‐based guides and 2.8 mm and 4.9° for sCT‐based guides. More specifically, there were no intermodal statistically significant differences in the guides positioning. In addition, the intermodal rotational and translational limits of agreements were within the interobserver limits of agreement, suggesting the interchangeability of CT and sCT for the design of guides for long bone osteotomy. As an example, Fig. 6 shows saw guides positioning differences between CT and sCT.

In the skull, deviations of ±1.4 mm were reported between BB‐MR and CT segmentations.^63^ The surgical guides resulting from the corresponding bone models were positioned on the skull with errors within ±0.6 mm for CT‐guides and ±0.8 mm for MR‐guides relative to their respective virtual planning. Given such differences, the average deviation from planned postoperative craniofacial reconstruction was within ±1.3 mm when using CT‐based guides and ±1.5 mm when using BB‐MRI‐based guides, with no statistical differences between the two modalities.^63^

## MRI for Diagnosing Bone Pathologies

Bone visualization and diagnosis on MR images can be hampered by the presence of water–fat interfaces, specific soft tissues like tendons, or air pockets in the vicinity of the bone since they may share the same low signal (Fig. 4). Therefore, the advantages and challenges of employing MRI for diagnosing bone‐related pathologies are anatomy‐specific. Multiple regions, including the skull, spine, shoulder, and pelvis have been assessed in recent years and are discussed in this section. For each anatomical region, the focus has been placed on two aspects: the potential of MRI for 1) detecting structural changes and for 2) measuring morphometric parameters of the bone. Structural changes include the detection of fractures, bone erosion, or sclerosis. On the other hand, morphometric parameters offer a quantitative assessment of bones which provides a standardized discrimination between “normal” and pathological regions and can influence therapeutic decision‐making.

### Skull

The development of MR protocols for skull visualization was favored by the routine acquisition of MR images for a wide range of clinical indications. In the standard of care, CT is indicated for trauma patients and for detecting osseous lesions, whereas MR images can be acquired for the detection of intracranial pathologies such as hemorrhage, ischemic changes, tumor, or other neurological disorders.^1^, ^2^, ^52^, ^53^, ^57^, ^70^

#### STRUCTURAL CHANGES

Skull composition and anatomy vary between stages of life, resulting in an age‐dependent diagnostic power of MRI and CT. Infants under 6 months have a thin skull (~1 mm thick) with high water content.^64^ In children under 2 years of age, sutures are wider^61^, ^83^ and harder to distinguish from trauma‐induced fractures.^61^, ^70^ Lastly, cranial sutures tend to be less conspicuous in adults than children on BB‐MRI^20^ and UTE‐MRI^54^ as compared to CT because of a lower suture‐to‐skull contrast in adults.

**FIGURE 7:**
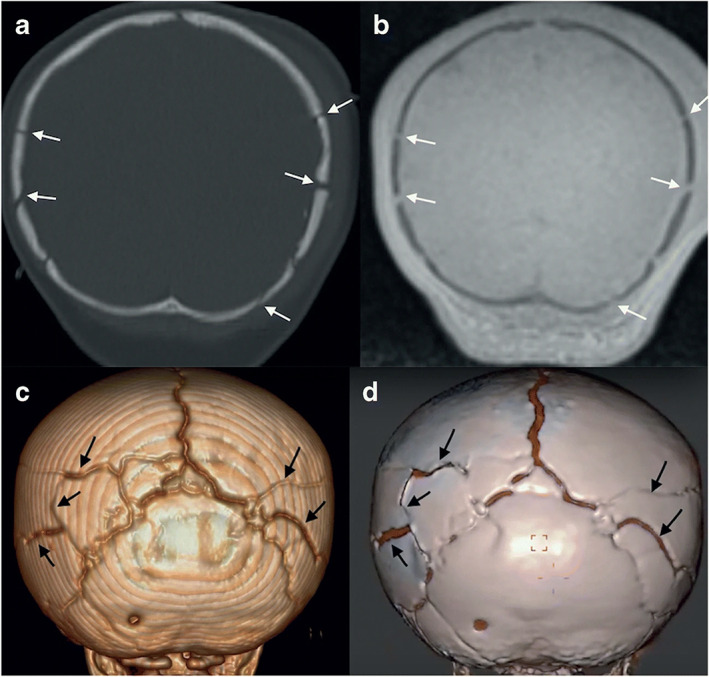
A 9‐month‐old with multiple skull fractures (arrows) demonstrated on coronal head computed tomography (CT) (**a**), and coronal black bone magnetic resonance imaging (MRI) (**b**) and the corresponding 3D rendering (**c**, **d**). Reprinted by permission from Springer Nature from reference 70.

Nonetheless, the premature fusion of cranial sutures could be evaluated in infants and children with good to excellent inter‐ and intraobserver variability.^20^, ^64^ For detecting skull fractures in children, BB‐MRI had an overall sensitivity of 66.7% and specificity of 87.5%, with errors originating from confusion between linear fractures and sutures, mainly in children under 2 years of age.^61^ However, the addition of BB‐MRI‐based 3D skull renderings increased the sensitivity to 83% and the specificity to 100% in a different cohort of patients under 30 months.^70^ Skull renderings obtained from MRI as shown in Fig. 7 were stated to be valuable for diagnosis in most patients,^70^ which is in line with results obtained in CT images.^83^ When UTE‐MR images were used, promising results were reported for the detection of fractures in patients aged from 1 month to 71 years.^52^ Sensitivity, specificity, and accuracy were all higher than 90%.^52^ The length and depth of the fractures could be measured on UTE‐MR images with no statistical difference compared to measurements made on CT images. For both UTE and BB‐MRI,^52^, ^70^ good to excellent inter and intraobserver agreement was reported for detecting fractures. Moreover, MRI could detect other pathologies such as edema, axonal injuries, and fractures accompanied by hemorrhages that were not visible on CT.^52^, ^61^

With regard to the temporomandibular joint and mandible, other structural differences were visible on UTE‐ and ZTE‐MRI with good to excellent inter‐ and intraobserver variability,^53^, ^57^ benefitting from a good UTE/ZTE‐to‐CT voxelwise intensity correlation in healthy and diseased bones.^21^, ^53^ ZTE‐MRI revealed flattening and osteophytes in the mandibular condyles with near‐perfect agreement to cone beam CT^57^ whereas intermodal agreement for detecting medullary sclerosis was excellent on ZTE and moderate on UTE images.^53^, ^57^ However, erosions, osteolysis, and periosteal reactions were more difficult to diagnose with only moderate intermodal agreement. In particular, periosteal reactions could be confused with air pockets on UTE images as both bone and air share similar intensities.^53^

Overall, all MR images suffered from misdiagnoses at interfaces between bone and air. Air and bone share similar intensities, making the bone/air interface difficult to distinguish.^52^, ^54^, ^61^, ^70^ Particularly difficult regions were the mastoid process,^52^, ^61^, ^70^ the paranasal sinuses,^52^, ^70^ and complex bone/fluid interfaces with high anatomical details like the inner ear.^58^ Such interfaces can cause misdiagnoses^58^, ^61^ and complicate automated processes for segmentation.^54^, ^87^ To facilitate the distinction between tissues and air, phase information could complement the magnitude images,^90^ although the processing of phase images is complex and can be error prone.^90^, ^91^

#### MORPHOMETRIC ASSESSMENT

Following the diagnosis, morphometric analysis of the mandible and cranium can be performed to plan craniofacial or maxillofacial surgeries.^54^, ^65^ However, the measurement of such local parameters on MR images was not consistent between studies.^54^, ^65^ On UTE‐based skull segmentations, intermodal differences of up to 2 mm and average deviation to cadaveric measurements of up to 4 mm were reported for eight anatomical parameters.^54^ By comparison, on SE‐derived images, performances were deemed statistically equivalent to cone beam CT, with average differences under 0.61 mm and 0.65° for 27 parameters^65^ and BB‐based bone segmentations deviated by ±1.4 mm from CT segmentations.^63^ Such differences might be due to differences in resolution as the voxel size was twice as small on T1w‐SE and BB‐MRI images as on UTE images (~0.5 mm vs. 1.1 mm).^54^, ^63^, ^65^ The T1w‐SE and BB acquisitions also had high receiver bandwidth (>610 Hz/px—Table 1) to maintain geometrical integrity.

### Spine

When imaging the spine, MRI is the modality of choice in many applications as it offers valuable information on the neural structures, the intervertebral discs, bone marrow, and the surrounding soft tissues.^17^, ^36^ CT is typically acquired to assess the osseous involvement of soft tissue pathologies, for assessing bony abnormalities such as fractures, spondylosis, spondylolysis, or for surgical planning,^5^, ^17^, ^39^ owing to the superior cortical bone contrast and to the isotropic resolution of CT that enables multiplanar reformatting.^5^

#### STRUCTURAL CHANGES

When patients are suspected of having a vertebral fracture, CT images are routinely acquired to depict the extent of fracture. In addition, CT imaging is preferred in patients for whom a quick assessment is required, eg, patients who suffered high‐velocity accidents. Additional MRI is sometimes acquired, mainly to rule out occult injuries and to identify spinal cord lesions.^4^ In this context, MRI can also aid in distinguishing acute from old fractures,^3^ and can help diagnose specific types of fractures, such as stress fractures.^39^ Diagnostic performance statistics for detecting acute or stress fractures on MRI were excellent for S‐GRE, VS‐GRE, and UTE images with specificity, sensitivity, and accuracy above 90% when CT was used as ground truth.^36^, ^37^, ^39^ The interobserver agreement was good to excellent^36^, ^37^, ^39^ and was comparable between CT and MRI.^37^, ^39^ For standard of care SE images, the specificity and accuracy for detecting fractures was above 95% while the sensitivity was 75% for incomplete fractures and 91% for complete fractures.^22^

**FIGURE 8:**
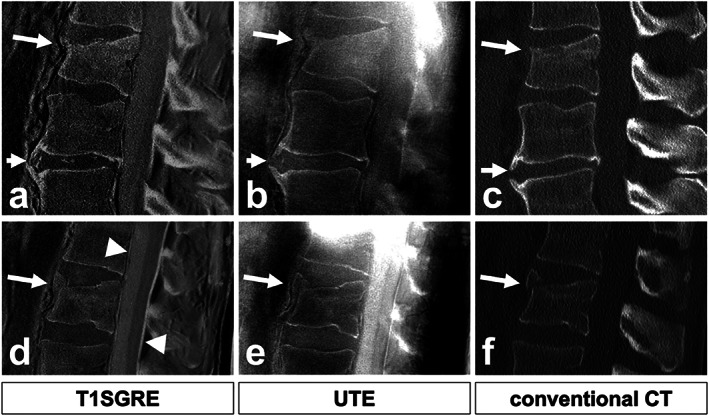
Comparison of T1SGRE‐derived CT‐like images (**a**, **d**), UTE images (**b**, **e**), and conventional CT images (**c**, **f**). In one patient (a–c), a wedge‐compression fracture of L1 with signs of an acute pathology such as a compaction zone can be depicted (upper arrows), as well as ventral and small dorsal osteophytes on level L2/3 (lower arrows). In another patient (d–f), another wedge‐compression fracture of L2 with a triangular teardrop‐like fragment can be identified (arrows). Also note the thin hyperintense line running longitudinally along the posterior walls of vertebral bodies representing the posterior longitudinal ligament as well as the thicker hyperintense line posterior to the dural sac representing the ligamenta flava (arrowheads; d), which are not depicted on CT (f), and must not be misinterpreted as ligament calcifications. Figure reproduced without modification from reference 36 under the Creative Commons Attribution 4.0 International License (http://creativecommons.org/licenses/by/4.0/). T1SGRE = T1 radiofrequency spoiled gradient‐echo; UTE = ultrashort echo time; CT = computed tomography.

Overall, GRE images, including S‐GRE and VS‐GRE seemed to outperform standard of care SE images for detecting fractures,^22^, ^37^ demonstrating a higher sensitivity in the delineation of the fracture line, probably owing to their thinner slices (see Table 1). Regarding S‐GRE and UTE images, differences are more questionable. In a cadaveric study,^37^ S‐GRE was reported as the most decisive standard of care sequence for detecting fractures in the pars interarticulares but observers were more confident in their diagnosis and missed fewer fractures when using UTE‐MRI. In particular, UTE imaging demonstrated a better interobserver agreement thanks to its CT‐like characteristics and a better contrast between the bone and fracture gap.^37^ On the other hand, in patients with suspected acute vertebral fractures, S‐GRE images outperformed UTE images in terms of the intermodal and interobserver agreements for detecting fractures, which was also the case for sclerosis, osteophytes, and joint degeneration.^36^ The difference in diagnostic quality between UTE and S‐GRE images between the cadaveric^37^ and in vivo^36^ studies might have several sources. In vivo, despite the radial k‐space sampling, the UTE images were reported to be prone to pulsation and motion artifacts^36^ that were not present ex vivo. In addition, the presence of multiple tissue types and air in the surrounding of the spine could result in susceptibility artifacts, especially seen in UTE images, more than GRE or ZTE images.^5^, ^36^ A comparison between S‐GRE, UTE, and CT images for the detection of acute fractures and osteophytes is given in Fig. 8.

SE, S‐GRE, VS‐GRE, and UTE sequences all misdiagnosed fractures in some patients,^22^, ^36^, ^39^ in part because of the misinterpretation of areas of bone sclerosis. These could be confused with subtle fractures or edema‐like changes,^36^, ^39^ or could mask fractures.^22^ However, the addition of a fluid‐sensitive MR sequence like short tau inversion recovery could reveal bone marrow edema and enable the detection of stress reactions that are invisible on CT images^22^, ^36^, ^39^ but can potentially change the patient's clinical management.^39^ For diagnosing sclerosis, GRE images seemed superior to UTE images^36^ or SE images as seen in the sacroiliac joint.^42^, ^92^

Other structural anomalies, including degenerative changes in the craniocervical junction^55^ and in the cervical spine^5^ that can cause neck pain were also investigated. In these cases, MRI is suitable for detecting ligamentous or intervertebral disc pathologies whereas CT can detect stenosis of the cervical spinal canal or neuroforamina.^5^ In both the craniocervical junction and cervical spine, degenerative changes were graded with good intermodal agreement,^5^, ^55^ similar to the interobserver agreement on CT.^5^ This was facilitated by multiplanar reformatting possible on isotropic ZTE images.^5^ Overall, good to excellent inter‐ and intraobserver agreement was reported with MRI.^5^, ^55^ However, when using MR images with inverted intensities, care needs to be taken not to misinterpret the apparent high signal intensity of ligaments, or of the gas accumulation in the intervertebral discs as calcifications^36^ as seen in Fig. 8.

#### MORPHOMETRIC ASSESSMENT

The diagnosis of degenerative changes can also be made quantitatively by measuring morphometric parameters including vertebral body and intervertebral disc parameters. Despite a good to excellent intermodal agreement in measuring vertebral body height on UTE, S‐GRE, and sCT images, and an excellent interobserver agreement,^36^, ^79^ the accuracy of morphological vertebral assessment was highly dependent on the MR acquisition. Average differences in the vertebral height of 0.26 mm were reported in the sCT^80^ with limits of agreement within ±2 mm for S‐GRE images^36^ and within 6–10 mm for UTE images.^36^ For intervertebral disc heights, limits of agreement of ±2–3 mm were reported between S‐GRE and CT images and of ±4 mm between UTE and CT images.^36^ Similarly, CT/UTE intermodal limits of agreements were within ±1 mm for the distance between the cranium and C1 and within ±2–4 mm between the cranium and C2.^55^ However, these intermodal differences in the distances between the cranium and cervical spine were not significant and may partly originate from the differences in resolution between MR (0.8 mm × 0.8 mm × 1.2 mm) and CT (<0.6 mm × 0.6 mm × 0.6 mm).

### Shoulder

In the shoulder, MR examinations are commonly performed to examine the ligaments, the rotator cuff, the labrum, and the joint capsule, eg, after shoulder dislocation.^7^, ^35^, ^93^ However, standard of care T1w‐SE images have similar low intensity for cortical bone and labrum,^40^ warranting a CT examination to assess the glenohumeral bone architecture and review bone changes. In particular, the amount of glenoid bone loss, and to a lesser extent humeral deformity, often associated with shoulder dislocation, determines the clinical management plan.^86^

#### STRUCTURAL CHANGES

On MR images acquired with^35^, ^40^ or without^94^ intra‐articular contrast injection, a strong correlation (*r* > 0.8) was found between MR and CT for the glenoid width and percentage bone loss. The mean glenoid bone loss error was under 2.5% for both modalities,^94^ with intermodal differences not statistically different.^35^, ^40^ Furthermore, MR and CT measurements had good correlation with arthroscopy as percentage bone loss differences under 3% were reported between MR and arthroscopy and under 1% between CT and arthroscopy.

When MR‐based 3D bone renderings were compared to CT, bone defects were also equivalently visible on the bone reconstructions^16^ with no statistically significant differences between MR‐ and CT‐based renderings,^7^, ^29^, ^86^ good to excellent intermodal correlation,^7^, ^86^ excellent intermodal agreement,^19^, ^26^ and submillimeter/<1% average defect size difference.^7^, ^19^, ^29^ Although small on average, some intermodal differences could reach up to 3 mm/10% difference in glenoid bone loss,^19^, ^86^ potentially influencing clinical management for a minority of patients. However, such large differences were not systematically reported, with some maximal differences within ±7.5%.^26^, ^35^, ^40^

In addition to diagnosing bone loss, MR was used to detect fractures in the humerus and scapula with good intermodal agreement, and excellent sensitivity and specificity (>90%)^40^, ^59^ using ZTE‐MR and VS‐GRE images. Fracture extent was measured equivalently on MR and CT.^16^ ZTE‐MR images were able to reveal bone depression, bone resorption, and bone fragments better than standard of care PDw images in most patients, along with a good ZTE‐to‐CT intermodal agreement for detecting bone fragments and osteoarthritis.^59^ Moreover, ZTE‐MRI surpassed CT in revealing cortical bone and intraosseous lesions within a single image.^59^ In particular, bone marrow edema and cysts that remained undetected in CT were visible on ZTE images. As cysts indicate regions of lower bone quality, it is important to accurately detect them, particularly when the images are used for guidance of surgical planning.^59^

#### MORPHOMETRIC ASSESSMENT

Treatment planning of shoulder instability might include the measurement of morphometric parameters of the glenoid on 2D images or 3D renderings. The glenoid morphometric accuracy on MRI was comparable to CT as demonstrated by an excellent intermodal agreement with no statistical difference in the measurement on glenoid vault^59^ and glenoid version angle,^59^, ^93^, ^95^ using ZTE^59^ or standard of care MR images.^93^, ^95^ Intermodal agreement was good when comparing certain shoulder‐specific parameters, with limits of agreements within 6 mm for measuring glenoid vault depth^59^ and within 5° for the version angle^59^, ^95^ for most patients. However, for some patients, these measurements could differ drastically due to blurring and reduced FOVs on the MR images.^59^, ^95^ The intermodal limits of agreements were within the interobserver limits of agreement.^59^

In a similar way, the geometrical accuracy of MR‐based 3D bone renderings was compared to CT. Measuring glenoid/humeral width, height, and surface areas was equivalent between CT and MRI, although some statistically significant differences could be found^16^ but not systematically.^7^, ^29^ In particular, the average intermodal differences in glenoid and humeral surfaces were within ±10%,^7^, ^29^ and in glenoid/humeral width and height were within ±1 mm.^16^, ^29^

Based on bone rendering and radiography, kinematic analysis of joints can also be performed to quantify changes to the joint position and contact points.^41^, ^46^ Hence, using S‐GRE or CT‐like VS‐GRE images, digitally reconstructed radiographs (DRR) have been generated and registered to radiography images for shoulder and knee kinematic analyses.^41^, ^46^ Registration errors and kinematics measurements errors were larger for MR‐based DRRs than CT‐based DRR and showed an RMSE under 2.2 mm and 2.6° (vs. 1.6 mm and 2.2° for CT).^41^, ^46^ Because CT and radiography share the same contrast mechanism, intensity‐based metrics can be used for the CT‐DRR to radiograph registration whereas only edge‐information was used for the MRI‐DRR to radiograph registration,^41^ potentially explaining the larger registration errors of MRI‐based DRRs.

**FIGURE 9:**
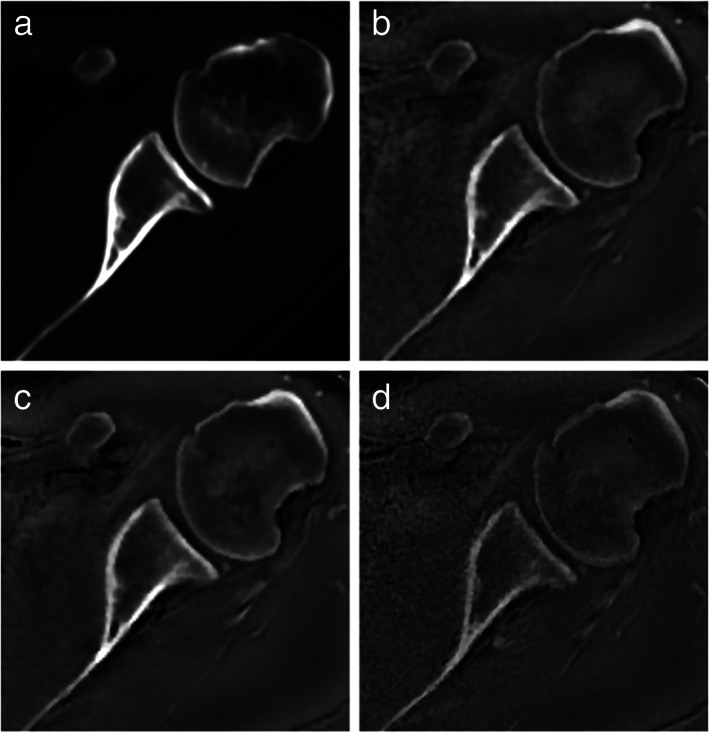
Axial computed tomography and zero echo time magnetic resonance imaging of left shoulder in a 38‐year‐old man. Axial images obtained by computed tomography (**a**) and zero echo time magnetic resonance imaging at 1.0 mm^3^ (**b**), 0.8 mm^3^ (**c**), and 0.7 mm^3^ (**d**) all show high‐contrast imaging of the osseous structures, including the glenoid and glenohumeral joint. Reprinted with permission from reference 19.

Overall, MR and CT showed good to excellent inter‐ and intraobserver variability in diagnosing bone pathologies or performing morphometric measurements in the shoulder,^19^, ^26^, ^35^, ^40^, ^93^, ^95^ with good to excellent intermodal agreement.^19^, ^26^, ^59^, ^86^ Figure 9 compares the CT image of a shoulder to the corresponding ZTE images acquired at different resolutions.

### Pelvis

The pelvic bone connects the upper body to the lower limbs through the sacroiliac and hip joints. Both joints can be subjected to degenerative osteoarthritic changes, affecting the bone and the surrounding soft tissues. In the sacroiliac joint, spondyloarthritis induces bone marrow edema and inflammatory lesions that can be detected with MRI, and structural lesions such as erosions, sclerosis, or ankylosis that may be detected with MRI,^96^, ^97^ but are better defined on CT.^6^, ^97^, ^98^ In the hip joint, hip dysplasia and femoroacetabular impingement are morphological hip conditions that affect bone, and soft tissues including but not limited to cartilage and labrum. For these conditions, clinical care usually includes morphometric assessment of the joint made on radiograph, with optional addition of CT or MRI for diagnosis, a soft tissue evaluation with MRI and a bone rendering based on CT for surgical planning. To limit adverse ionizing radiation, MR has been investigated as a diagnostic tool for detecting structural bone lesions and performing bone morphometric assessments and 3D renderings.

#### STRUCTURAL CHANGES

**FIGURE 10:**
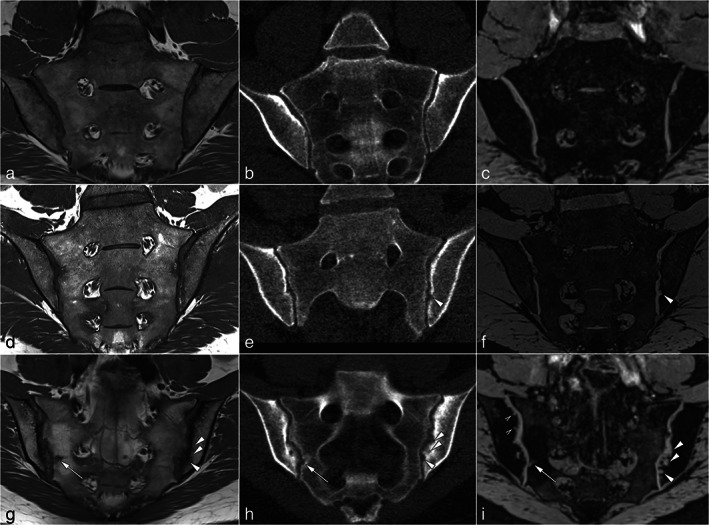
Imaging examples. (**a**, **d**, and **g**) Oblique coronal MR‐T1 sequence; (**b**, **e**, and **h**) low‐dose CT images in oblique coronal reconstruction; (**c**, **f**, and **i**) oblique coronal MR‐VIBE sequence. Slice positions and orientation are identical for T1 and VIBE. Low‐dose CT was reconstructed to match orientation and position. (a–c) normal findings in the sacroiliac joint without erosions. (d–f) Patient with axial spondyloarthritis with a single but prominent erosion of the left iliac surface that is shown by low‐dose CT and MR‐VIBE (arrowheads) but not by MR‐T1. (g–i) Patient with axial spondyloarthritis and multiple erosions. Some erosions (arrow) are depicted by all modalities. However, some larger erosions are hardly seen with MR‐T1 due to sclerosis, while they are more conspicuous using low‐dose CT and MR‐VIBE (arrowheads). The smallest erosions are only depicted with MR‐VIBE (open arrowheads). MR = magnetic resonance; CT = computed tomography; VIBE = volumetric interpolated breath‐hold examination. Reprinted with permission from reference 42.

In the sacroiliac joint, SE, GRE, and sCT images were used to assess structural changes. T1w‐SE images were shown to strongly correlate with low‐dose CT for detecting erosions and were able to reveal 88% of erosions.^92^ However, standard MRI missed some cases of axial spondyloarthritis when used alone.^92^ On more dedicated images to visualize bone, including VS‐GRE or sCT images, higher diagnostic accuracy and diagnostic confidence were achieved for detecting erosions,^6^, ^42^, ^78^ especially when sclerosis was present.^42^ In particular, the sensitivity and specificity for detecting erosions increased between standard and dedicated images, reaching a sensitivity above 70%,^6^, ^42^, ^78^ a specificity around 90%,^6^, ^42^, ^78^ and an accuracy above 90%.^78^ A qualitative comparison between T1w, VS‐GRE, and CT images for diagnosing erosions is presented in Fig. 10.

Standard MR images were also able to detect 92% of joint space alterations, and to a lesser extent sclerosis,^92^ while sCT could diagnose sclerosis and ankylosis with accuracies higher than 90%.^78^ Dedicated MR imaging was also reliable owing to a good to excellent interobserver agreement^6^, ^78^ and repeatable with good intraobserver agreement,^78^ in accordance with CT imaging.^6^, ^78^ Moreover, observers were more confident when scoring VS‐GRE images than low‐dose CT images because of the noise of low‐dose CT^42^ and equally confident when scoring sCT compared to CT images.^78^ Some erosions were only visible on dedicated MR images and not on CT images,^6^, ^42^ especially in young patients^6^ and for small lesions.^42^ As no erosions were detected in healthy controls,^42^ this suggests that the observed destructive changes were no artifact and that MR was superior in revealing those erosions.

In addition, MR images have been acquired to describe fractures in the hip joint. MRI has been shown to perform better than CT for the detection of fractures in the hip in elderly patients,^99^, ^100^ but also in adolescents^101^ and children.^101^, ^102^, ^103^ All fractures detected on CT were also detected on MR^100^, ^101^ and some fractures were visible on MR but not CT,^101^ or misdiagnosed on CT resulting in changes in the clinical management plan,^100^ especially regarding instructions for weight bearing. In patients under the age of 13 years, the posterior acetabular wall is not fully ossified^103^ and the MRI findings of traumatic hip dislocations with acetabular fractures were better correlated with intraoperative findings than CT findings,^102^ which did not always directly detect acetabular fractures.^103^ Some soft tissue defects, oblivious to CT, were also identified on MRI. These included entrapment of labra and posterior acetabular cartilage fractures. The detection of entrapment of labra, in particular, had an influence on the patient clinical management.^101^

#### MORPHOMETRIC ASSESSMENT

The measurement of morphometric parameters is especially important in the hip joint for the diagnosis of hip dysplasia and femoroacetabular impingement and has been investigated on VS‐GRE,^43^, ^45^ ZTE,^23^ sCT,^77^ or intermediate‐weighted images.^43^ All imaging techniques found good to excellent intermodal agreement for measuring the acetabular version^23^, ^43^, ^45^, ^77^ with excellent inter‐ and intraobserver agreements^23^, ^45^, ^77^ and statistically equivalent measures given an acceptable error below 4.3°.^45^, ^77^ The reported limits of agreements were, however, mixed. On VS‐GRE and sCT images, intermodal limits of agreements were in line with the intraobserver variability, within ±4.2°.^45^, ^77^ On ZTE images, on the other hand, intermodal limits of agreements of acetabular version reached 11.3°, higher than the 8° obtained for the interobserver variability on CT.^23^ These differences might originate from the fact that pelvic tilt was not standardized in the ZTE study.^23^ Other parameters that were compared include the lateral center edge and alpha angles. The intermodal agreement was good to excellent^23^, ^43^ with intermodal limits of agreements roughly within 12°^23^, ^43^, ^77^ and bounded by the interobserver limits of agreement achieved on CT.^23^

Femoral parameters such as the femoral anteversion were also measured and compared between CT and MRI. In two studies using standard clinical sequences,^104^, ^105^ a strong correlation between CT and MR measurements was reported with correlation coefficients of 0.77 and 0.80 between the two modalities. However, the intermodal absolute agreement was poor with biases ranging from 5° to 10°, probably because of interscan positioning differences. The MR examination being long (30–45 minutes), patients might be given knee wedges^105^ to bend the knee, or can relax into greater external rotation of the hip, possibly explaining such differences. When measured in infants with developmental dysplasia of the hip^106^ on CT and T1w‐SE images, the intermodal, intraobserver, and interobserver agreements for the femoral version were all excellent (intraclass correlation coefficient >0.9), demonstrating the reliability and reproducibility of the methods. In such a young population, MRI had the advantage over CT that it was able to visualize the not fully ossified femoral condyle in infants under the age of 6 months. In these cases, the condylar plane could be defined more accurately on MR than CT.

**FIGURE 11:**
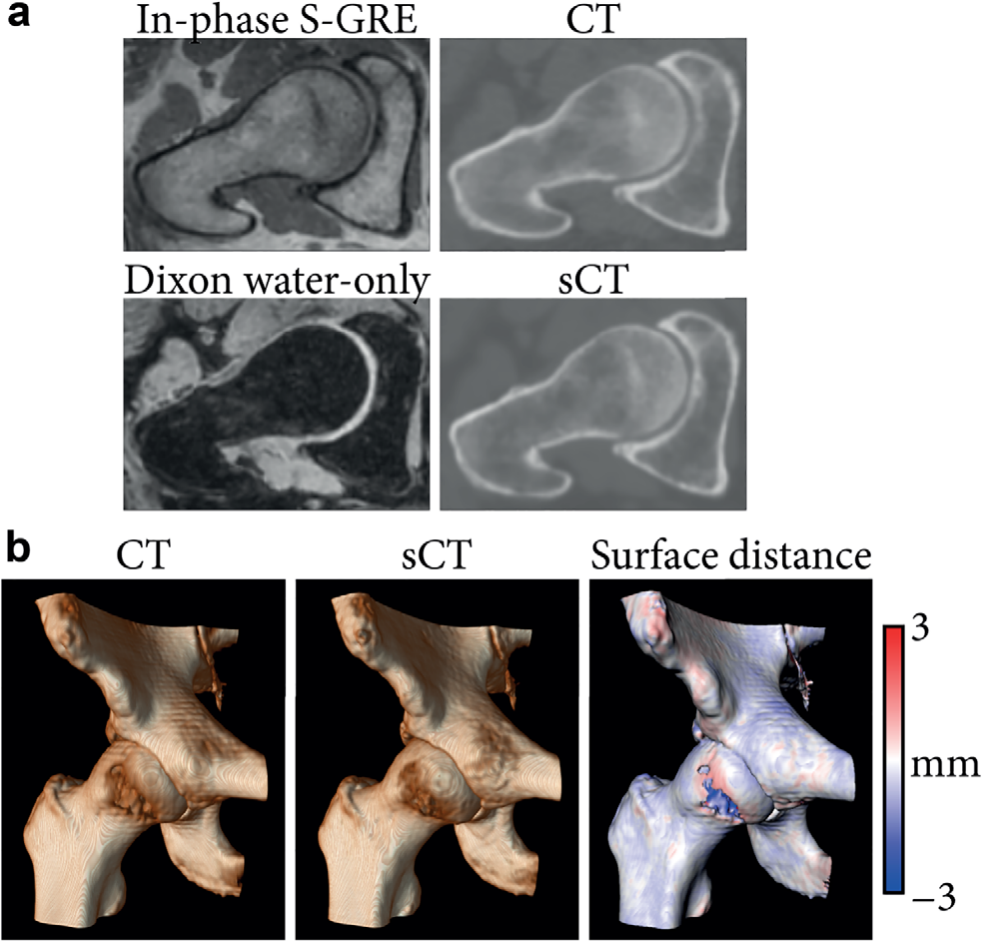
Magnetic resonance imaging (MRI)‐ and computed tomography (CT)‐based hip imaging. (**a**) Radial reformats of in‐phase radiofrequency spoiled gradient‐echo (in‐phase S‐GRE), Dixon reconstructed water‐only, CT, and synthetic CT (sCT) images. (**b**) Bone renderings obtained by applying a 150 Hounsfield unit threshold on CT and sCT images of a hip joint. Surface distance between the CT‐ and sCT‐based renderings was computed and mapped on the CT‐based rendering. Negative values indicate the CT is larger.

Using bone renderings, the measurement of local morphometric parameters, including the center‐edge angle and acetabular version was similar between CT and MR, with average intermodal differences under 4°.^28^, ^30^, ^44^ Hip range of motion measurements were also compared between CT and MR with average differences under 4° for all rotations and with limits of agreements within ±6°.^30^ All measurements had excellent intermodal correlation, intermodal agreement, and interobserver agreement.^30^, ^44^ Correspondingly, such models were able to diagnose femoroacetabular impingement or hip dysplasia with 100% agreement reported between MR and CT for the presence and location of cam deformity,^28^ and good to excellent intermodal and interobserver agreements.^43^ Figure 11 presents 3D bone reconstructions as obtained from CT and sCT.

## Remaining Challenges

Overall, the use of MRI as a radiation‐free alternative to CT for bone visualization has received a lot of attention in the last decade and has been valued by multiple editorials^107^, ^108^, ^109^ and overviews.^110^, ^111^ Although promising, MRI does suffer from challenges related to data acquisition and accessibility to novel technologies. This section describes these challenges and discusses how they might affect the adoption of MRI for bone imaging in clinical practice.

### Challenges in the Acquisition

When planning an MRI scan, a trade‐off has to be made between the FOV, the resolution, the signal‐to‐noise ratio (SNR), and the acquisition time. The MRI sequences used for bone visualization pose different constraints to this trade‐off which may limit their applicability in specific situations. These constraints are related to several external factors, including the size of the region to be imaged, or also the intrinsic tissue‐specific factors like magnetic susceptibility. In this section, we address these aspects in relation to their use for bone visualization.

#### FIELD OF VIEW

In general, a limited FOV is chosen when planning MRI acquisitions to reduce scan time. However, some sequences like UTE or ZTE have spatially nonselective excitations which induce large fields of view and reduce their flexibility regarding other acquisition parameters (eg, spatial resolution) to keep a reasonable scan time.

For other sequences, the freedom to reduce the FOV has two constraints. First, the FOV must be large enough to make a proper diagnosis. This includes the visualization of landmarks for post‐acquisition image standardization (eg, correction of the anterior pelvic plane for hip imaging) and of a sufficiently large region for the measurement of morphometric parameters (eg, scapula for measuring the glenoid version in the shoulder^95^ and femoral shaft and condyles for measuring the femoral neck shaft angle^23^, ^43^). Second, care needs to be taken to avoid the edge of the FOV where the lower signal and field inhomogeneity may compromise the measurements.^33^ As a solution, MR images can be acquired in multiple blocks, overlapping or not, to obtain the necessary information.^18^, ^24^, ^30^, ^31^, ^32^ Such multi‐station acquisitions are, however, susceptible to slight changes in position between individual acquisitions that can compromise the geometric integrity of the bone.^24^

#### SPATIAL RESOLUTION

Image resolution was often lower on MRI than on CT, with voxel sizes usually ranging between 0.6 mm and 1 mm in the reviewed literature (see Table 1). Note that some of the reported resolutions are reconstructed resolutions and not acquired resolutions. Low‐resolution images induce more partial volume effects that can mask^54^ or, on the contrary, enlarge structures of interest, potentially resulting in the under‐ or over‐segmentation of bone on MR images.^58^ Furthermore, for 3D bone modeling, low resolutions result in high interpolation uncertainty and can cause stair‐step artifacts.^47^, ^59^, ^112^ However, increasing the resolution is not always beneficial as it is accompanied by a decrease in SNR or an increase in acquisition time without necessarily improving the diagnostic capabilities of the images.^19^, ^36^, ^38^

MR images usually had an (almost) isotropic resolution in the literature assessed for this review (Table 1). The voxel isotropy enables multiplanar reformatting of the images for an improved visualization of the vertebrae, of the glenoid, or of the femoral neck for the measurement of morphometric parameters in these regions. In addition, for 3D bone modeling, voxel isotropy makes the interpolation uncertainty equal in all directions, facilitating bone models interpretation and the subsequent modeling of surgical tools.

#### ACQUISITION TIME

Long acquisition times are problematic as they induce higher costs and potential motion artifacts. In children, in particular, motion artifacts could compromise the diagnostic quality of the MR images.^20^, ^70^ Voluntary motion in the youngest patients can be avoided, by either using immobilization,^70^ or sedation which includes the feed‐and‐sleep method^64^ or general anesthesia.^61^, ^64^ Although also sometimes required for CT acquisition, deeper sedation is usually needed during MRI acquisition because of the longer acquisition time. The use of anesthesia is however not risk‐free, especially on repeated occurrences.^113^ In addition, uncontrolled motion was seen in the spine,^36^ and the jaw,^53^ and is common in clinical care of the shoulder, weakening the diagnostic power of MRI. Nonetheless, motion artifacts can be reduced by using motion insensitive acquisition methods, including breath holds,^67^ interleaved scanning, increased parallel imaging with higher signal averaging, or radial sampling of the k‐space.^21^, ^55^, ^64^, ^70^

#### SUSCEPTIBILITY ARTIFACTS

MRI can also be impaired by magnetic susceptibility‐related distortions due to the shape of the body, or the presence of air or of implanted devices. Areas of concern for such artifacts are the spine, where the bone is surrounded by multiple magnetically differing soft tissues, air and/or metal instrumentation, the jaw which can contain orthodontic devices,^53^, ^65^ but also long bones with screw fixations^30^ or the skull with ventriculoperitoneal shunts.^61^ When expected, susceptibility artifacts can be partly mitigated by choosing the adequate MR sequence and acquisition parameters. At equivalent acquisition parameters, GRE sequences are more prone to susceptibility artifacts than SE sequences, and sequences such as UTE are more prone to field inhomogeneity artifacts than S‐GRE^36^ or ZTE.^5^ The geometrical distortions induced by susceptibility artifacts can be mitigated by increasing the receiver bandwidth at the cost of SNR, by applying the scanner's built‐in distortion correction, or by limiting the FOV around the scanner's isocenter, but they are never completely removed.^66^ In addition, although 3 T acquisitions are usually equivalent or better than 1.5 T acquisitions for bone visualization and segmentation,^43^, ^114^ lower field acquisitions should be favored when inhomogeneity artifacts are expected.^36^, ^61^ Low‐field MRI (<0.5 T) in particular could be acquired to diagnose pathologies associated with orthopedic hardware,^115^ given the assumption that low‐field MRI is not overly impacted by susceptibility artifacts and is able to image soft tissues in the vicinity of the implant. Other advantages of low‐field MRI include its low cost (purchase and maintenance), and, when considering musculoskeletal radiology, the ability to scan in weight‐bearing position.^115^ This, however, comes at the expanse of SNR and resolution.

### Challenges in MRI Access

#### MR CONTRAINDICATIONS

Compared to CT, MRI suffers from a multitude of contraindications that make it unavailable for some patients. For trauma patients, the access to an MRI can be limited by obstacles related to diagnostic speed, transport of the patient to the MRI, MRI incompatibility with life‐support or monitoring equipment, and patient implants. Metallic MR‐compatible devices are problematic when in the vicinity of the region of interest as they can generate susceptibility artifacts hampering the diagnosis. Devices that are not MR‐compatible, including some pacemakers and cochlear implants, preclude any MR acquisition. In addition, claustrophobic patients or patients unable to stay motionless might require sedation to undergo MRI, complicating the workflow, potentially causing adverse effects,^113^ and hindering compliance with the breathing instructions required for some sequences. Overall, in an emergency department, more than a quarter of the elderly patients coming after a trauma could have at least one contraindication for MRI.^99^

#### AVAILABILITY

Another issue of MRI is its availability for acquisition. CT being faster, it is more accessible, especially in cases of emergency.^70^, ^99^ When an MR system is acquired, the choice of the MR sequence might be driven by the available hardware. The sequences described in this review are not commonly present on all scanners. Dixon reconstruction is now usually built in the scanner,^68^ but sequences like UTE and ZTE might require modern hardware or specific chargeable licensing. They tend to be increasingly available and offered as standard sequences^19^ but as an example, all ZTE images presented in this review were obtained only on GE scanners. Tools for sCT generation from GRE‐derived images are also becoming commercially available.^78^

## Discussion

CT is considered the modality of choice for visualizing cortical bone in 3D. However, its adverse radiation burden^8^ has motivated the research into alternative modalities with lower radiation doses, including radiography‐based,^116^ low‐dose CT‐based,^117^ or MR‐based methods. In this competition, despite some challenges in the acquisition, MRI has favorable properties including its superior soft tissue contrast that can be exploited to concurrently assess the soft tissue involvement of musculoskeletal pathologies without the need for image registration, and the complete absence of ionizing radiation.

The utility of MRI as an imaging modality for visualizing bone has been shown in many areas of the human body. Overall, the CT‐to‐MR intermodal agreement for the diagnosis of osseous pathologies and for the measurement of anatomical parameters was good to excellent with multiple reports of statistical equivalence.^16^, ^45^, ^65^, ^77^ In addition, MRI could provide 3D bone renderings, critical in the clinical care for the skull, shoulder, or hip, with a submillimeter accuracy compared to CT, although in general representing an underestimation of the actual bone size.

MRI presented several advantages compared to CT in the diagnosis of musculoskeletal pathologies. First, immature bone as seen in the femur and pelvis of young children was better visualized on MR than on CT images.^101^, ^106^ Second, MRI can acquire soft tissue and bone information in a single examination. Sequences like VIBE, UTE, or multi‐echo steady state (MESS) can provide bone structural information while providing complementary information on other tissue, including cartilage.^118^, ^119^, ^120^, ^121^ This can promote joint biomechanical and kinematic modeling by limiting the need for registration.^18^, ^32^, ^122^ Some of the dedicated images also revealed fractures and lesions, like cysts or edema, that can improve patients' clinical management but which were not visible on CT. Furthermore, MRI can be used to generate simulated radiographs with a diagnostic quality similar to CT for imaging bone tumors, while providing additional information on tumor architecture and soft tissue extension.^48^ Lastly, MR sequences for imaging bone can also be combined with other sequences for specific imaging such as venous^64^ or fluid‐sensitive^36^, ^39^ imaging, magnetic resonance angiography, or quantitative susceptibility mapping^123^ for a more comprehensive diagnosis within a single modality.

The use of an MR‐based bone visualization could in the future be extended to facilitate clinical care motivated by the benefits of CT/MR fusion. Such fusions could be useful for the design of patient‐specific implants by combining bone and joint capsule information^124^ and have proven their potential for diagnostic and treatment purposes, by easing the diagnosis for junior readers,^125^ and by facilitating treatment guidance^9^, ^126^ and surgical navigation.^49^, ^50^, ^127^, ^128^ However, fusing MR and CT requires an intermodal registration that is not necessarily straightforward. With MR providing a CT‐like visualization of bone, visualization of soft tissues and bones can be obtained in one scanning session with similar body geometry, offering new perspectives for diagnosis, treatment planning, and guidance. Fusion of MRI with radiography has also been performed for kinematic analysis.^41^, ^46^

Is one sequence better than the other? VS‐GRE and ZTE sequences seemed to stand out with validation in multiple anatomies, owing to their rather fast acquisition offering isotropic images with good cortical bone‐to‐bone marrow and cortical bone‐to‐muscles contrasts that facilitate bone segmentation. VS‐GRE sequences are also robust to respiratory motion through breath holds while ZTE acquisitions are robust to motion in general thanks to their radial k‐space sampling. In parallel, sCT is gaining interest for orthopedics^77^, ^78^, ^82^ building upon its CT‐like HU, although care still needs to be taken in interpreting such artificial intelligence‐based images. In addition, the validity and robustness of single sCT generation models need to be carefully assessed across multiple MR vendors and sites. In general, awareness of the possible artifacts and MR image specificities, especially regarding air, ligaments, tendons, or water/fat interfaces, is required for all anatomical regions and MR sequences.^5^, ^20^, ^30^, ^36^, ^57^, ^61^, ^81^ However, getting acquainted with the use of MR images for measurements and diagnosis might be easier and faster on images with a CT‐like contrast, like ZTE or sCT, that have a high correspondence to CT images.^21^, ^27^, ^53^, ^80^ As of now, only a few studies compared multiple MR sequences^6^, ^22^, ^36^, ^37^, ^38^, ^42^, ^55^, ^78^ with equivalent acquisition parameters to CT, complicating definite conclusions, which might be specific to an anatomical region.

To conclude, MRI is a promising radiation‐free alternative to CT for the diagnosis and treatment planning of bone pathologies. The recent advances in hardware and software provide MR images with a spatial resolution and contrast that are similar to CT images for the detection of structural and degenerative bone changes. MRI will probably not replace CT for all its applications in the near future, especially not in emergency settings. However, for clinical indications where both bone and soft tissue information are required, these new approaches open new perspectives for comprehensive protocols that facilitate bone and soft tissue visualization and fusion, for diagnosis, treatment planning, and surgical guidance.
